# Numerical Study of Ultra-Broadband Metamaterial Perfect Absorber Based on Four-Corner Star Array

**DOI:** 10.3390/nano11092172

**Published:** 2021-08-25

**Authors:** Yu Cheng, Min Xiong, Ming Chen, Shijie Deng, Houquan Liu, Chuanxin Teng, Hongyan Yang, Hongchang Deng, Libo Yuan

**Affiliations:** Guangxi Key Laboratory of Optoelectronic Information Processing, Guilin University of Electronic Technology, Guilin 541004, China; chengyu@guet.edu.cn (Y.C.); shijie.deng@guet.edu.cn (S.D.); liuhouq@guet.edu.cn (H.L.); xinchuanteng@126.com (C.T.); yhy.gl@126.com (H.Y.); hcdeng@guet.edu.cn (H.D.); lbyuan@vip.sina.com (L.Y.)

**Keywords:** plasma, perfect absorber, ultra-wideband, metamaterial

## Abstract

In recent years, research on solar absorbers provides a significant breakthrough to solve the energy crisis. A perfect solar absorber based on a four-corner star array is designed and the absorption performance is analyzed numerically. The results show that the absorber reaches more than 90% of the full band in the range of 400–2000 nm. In particular, the absorption efficiency of the continuous more than 95% of the bandwidth reached 1391 nm, and the average absorption efficiency of the whole study band is more than 98%, and the loss of the solar spectrum only accounted for 2.7%. At the same time, the absorption efficiency can be adjusted by changing the geometric structure of the absorber. In addition, due to the perfect symmetry of the structure, it has an excellent insensitivity of the incident angle and polarization angle. In general, the proposed solar absorber has exciting prospects in solar energy collection and utilization, photothermal conversion and other related fields.

## 1. Introduction

The performance metamaterials are widely used in various fields due to their unique electromagnetic properties, such as metamaterial lenses [1,2,3], invisibility cloaks [4,5], surface sensors [6,7,8,9], wave absorbers [10,11,12,13,14,15], energy harvesters [16], etc. It is especially noteworthy that the energy crisis has brought new challenges and opportunities to all countries around the world [17,18], and solar energy has quickly won the favor of researchers because of its unique advantages. The research data show that the spectrum of solar energy radiation to the earth’s surface is mainly distributed in the range of 295–2500 nm, and nearly half of the energy is mainly concentrated in the visible region [19]. Therefore, it is urgent to design a device for broadband and efficient absorption of solar energy spectrum.

Up to now, the research on solar absorbers has mainly been focused on the joint design of material and structure. In terms of materials, the imaginary part of the metal determines the loss of incident light and the ability of plasma resonance. Meanwhile, the absorber should adapt to various special environments such as high temperature, which makes a class of metals with large constant imaginary part and high melting point, such as Ti and W, stand out from other traditional metals [20]. Compared with traditional metals such as gold and silver, which have plasmon resonance that can only work in a narrow band range, refractory metals have better plasmon properties and can improve the absorption efficiency in a wide frequency range due to their inherent losses [21]. In terms of structure design, the absorbers studied so far can be mainly divided into three types. The first type mainly refers to the simple structure preparation and the realization of broadband absorption within a certain range, such as a structure composed of periodic arrangement of titanium and silicon cubes and aluminum film [22], a periodic array based on Ti ring [23] and a multi-port ring based on two materials consisting of Ti and TiN [24], etc. However, the drawback is that the absorption bandwidth is narrow, and the absorption efficiency of the continuous over 90% absorption wavelength can only reach around 1300 nm. The second type mainly refers to the realization of a large absorption bandwidth of the absorber, such as a four-layer ring-disk structure (SiO_2_–W-SiO_2_–W) [19] or an elliptical titanium nanodisk array based on a silica–titanium–silica–titanium four-layer structure [25], but unfortunately this type of absorber has a low absorption rate in the visible light region. The broadband absorption is mainly due to the broadening of the absorption in the near infrared band, while nearly half of the energy in the solar spectrum is concentrated in the visible light region. The last one can better solve the problems of a narrow absorption bandwidth and low absorption efficiency in the visible light region, such as a quadruple staircase structure [26] or a stack structure consisting of multiple layers of disks of different sizes [21]; although this kind of structure achieves good absorption efficiency, it mainly relies on the complex structure, which will directly lead to the problems of high precision, difficulty and high cost of the preparation process. The wide spectrum absorption generated by the above three types of absorbers is mainly due to the novel structural design and the use of high melting point materials. In general, it is due to the continuous exploration of researchers that the study of absorbers is flourishing.

Therefore, the main problem to be solved in this paper is to design an absorber that perfectly combines the advantages of large absorption bandwidth, high absorption efficiency in visible light region, simple structure and low preparation cost. For this purpose, we proposed a four-corner star nanoarray, in which the novel design of the polygonal star greatly enhances the absorption of the solar spectrum, and the use of high melting point materials also makes the absorber adaptable to a variety of environments. Numerical analysis results further show that the absorption efficiency of the structure is not only more than 90% in the whole band, but also that more than 95% of the bandwidth reaches 1391 nm. More prominent is that the average absorption efficiency of the structure reaches 98%, and the solar energy loss of the whole band only accounts for 2.7%. The perfect symmetry of the structure also makes it insensitive to the angle of polarization and incidence. The above results indicate that the absorber will have a promising future in the fields of solar energy absorption and utilization, photothermal conversion, etc.

## 2. Materials and Methods

**Figure 1:**
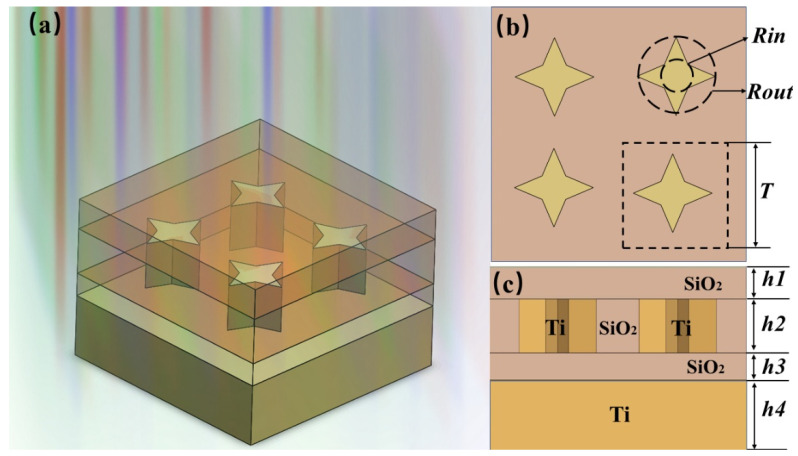
Schematic of the proposed nanostructure. (**a**) Three-dimensional diagram; (**b**) The X–Y plane graph; (**c**) The X–Z plane graph.

The structure diagram of the broadband absorber proposed in this paper is shown in Figure 1. Specifically, it is mainly composed of a four-corner star array in which the whole structure only uses two basic materials (silica and Ti) with high melting point properties, which greatly reduces the preparation cost and process difficulty. Both silica and Ti come from the materials database of FDTD software (FDTD Solutions 8.0, Lumerical, Vancouver, BC, Canada). The novel four-corner star structure is mainly constructed by the combination of the circumscribed circle and the inscribed circle. The four external vertex angles of the four-corner star are connected to the large circle with the radius of Rout, and the four internal vertex angles are connected to the small circle with the radius of Rin. The specific structure is shown in Figure 1b.

Figure 1 is a schematic diagram of the structure of the wide spectrum absorber designed in this paper. The meanings of the parameters in the structure are shown in Table 1.

In this paper, the finite difference time domain (FDTD) is used to simulate the structure. The 400–2000 nm plane wavelength spectrum light source is incident in the vertical direction. As for the boundary conditions, since the structure is in the form of an array, the X and Y directions are set as periodic boundary conditions. In the Z direction, the perfect absorbing layer boundary conditions are used, and the grid size is set as 3 nm × 3 nm × 3 nm. For the monitor, power detectors are placed above and below the structure to obtain the reflectivity *R* and transmittance *T* of the structure, and the absorption efficiency of the nanostructure is calculated by the formula A = 1 − *R* − *T*. The substrate h4 (composed of Ti) is not only used for physical support but is also thick enough to ensure that the light transmittance of the whole structure is almost zero. The equation [19] was shown as follows.

(1) $$\eta =\frac{\int_{\lambda_{\min}}^{\lambda_{\max}}(1-R(\omega )-T(\omega ))*I_{\mathrm{AM}1.5}(\omega )*d\omega }{\int_{\lambda_{\min}}^{\lambda_{\max}}I_{\mathrm{AM}1.5}(\omega )d\omega }$$

In the above equation, $I_{\mathrm{AM}1.5}(\omega )$ is the energy corresponding to the standard solar spectral data, R(ω) is the reflectivity and T(ω) is the transmittance. The absorption efficiency was obtained by Equation (1).

**Figure 2:**
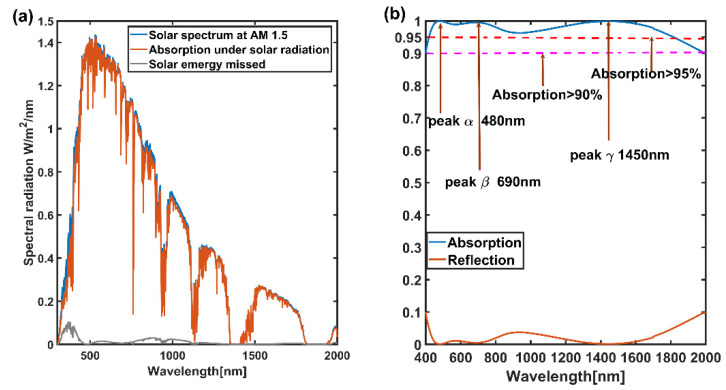
Diagram of optimal absorption efficiency. (**a**) Actual absorption graph of solar energy spectrum; (**b**) Absorption and loss efficiency graph.

By optimizing the structure of the absorption of the related parameters to get optimal results, as shown in Figure 2, specifically, the geometrical parameters of the structure under the optimal results are as follows: *h1* = 80 nm, *h2* = 90 nm, *h3* = 46 nm, *h4* = 300 nm, *Rout* = 110 nm, *Rin* = 45 nm, *T* = 240 nm. At the same time, in order to further demonstrate the actual absorption of solar spectrum, Figure 2a shows the actual solar spectrum energy and absorb solar energy spectrum diagram. It can be seen that the overall absorption efficiency of the absorber performance is perfect, and the energy of the loss spectrum is very small; at the same time, the absorption efficiency curve shows three absorption peaks, namely peak α, peak β and peak γ, respectively. The corresponding peak wavelengths are 480 nm, 690 nm and 1450 nm, respectively.

## 3. Results and Discussion

**Figure 3:**
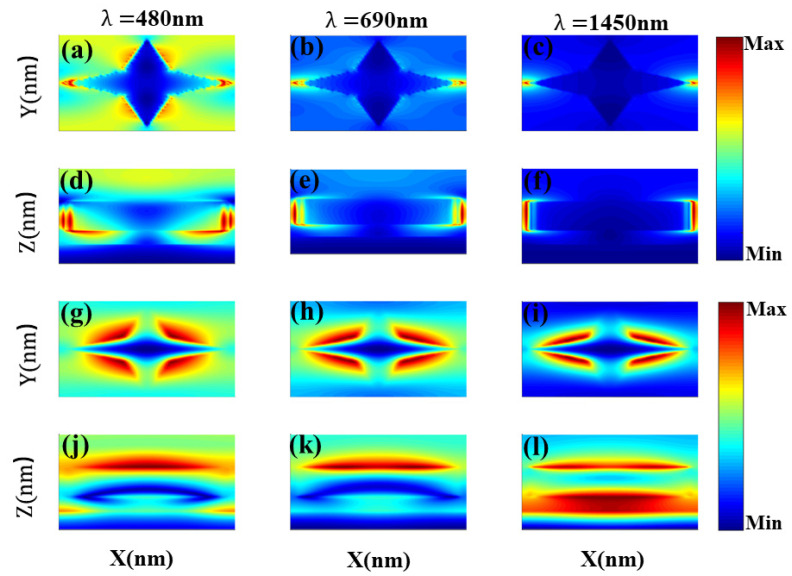
(**a**–**c**) distributions of the electric field |E| (color bar in the X–Y plane) and (**d**–**f**) electric field |E| (color bar in the X–Z plane), (**g**–**i**) distributions of the electric magnetic |H| (color bar in the X–Y plane) and (**j**–**l**) magnetic field |H| (color bar in the X–Z plane); the corresponding wavelengths of each column of images are 480 nm, 690 nm and 1450 nm, respectively.

Benefiting the absorption mechanism of the broadband absorber proposed in this paper can be summarized as the result of localized surface plasmon resonance, strong plasma near-field coupling, Fabry–Perot resonance and the unique properties of high-loss materials [19,24,25,27,28]. In order to better explain the internal mechanism causing wide spectrum absorption, this paper further studies the electric field intensity distribution diagram and the magnetic field intensity distribution diagram corresponding to three absorption peaks, as shown in Figure 3, in which (a)–(f) are the electric field diagrams in X–Y plane and the X–Z plane, and (g)–(l) are the magnetic field diagrams in X–Y plane and the X–Z plane.

For λ = 480 nm corresponding to peak α, it can be seen from Figure 3a that the tip of the four-pointed star presents a strong electric field, which means that the plasma near-field coupling between the metal arrays is excited. Moreover, the distribution of the strong electric field around the side of the four-pointed star and the diagrams (g), (j) show the magnetic field mainly concentrated in the vicinity of the four-pointed star and the upper surface. These can be regarded as the local surface plasmon resonance of the metal surface. At the same time, it can be seen from Figure 3d that there is a strong electric field between the four-pointed star and the bottom layer, which is mainly attributed to the fact that the four-corner star array, the bottom layer of Si0_2_ and the base layer Ti jointly form a Fabry–Perot cavity with a low quality factor, leading to the wide-band absorption resonance caused by the resonance of the F–P cavity [22]. To sum up, the absorption peak at 480 nm is mainly acted on by three mechanisms. For λ = 690 nm corresponding to peak β, the corresponding electric field diagrams (b), (e) and magnetic field diagrams (h), (k) are all compared with the electric field diagrams (a), (d) and magnetic field diagrams (g), (j) corresponding to λ = 480 nm, showing a weakening trend. The strong electric field near the four-pointed star in diagrams (b) disappears, which indicates that the broadband absorption at this wavelength is mainly caused by the near-field coupling of plasmons between the metal arrays and the Fabry–Perot (FP) cavity excitation. For λ = 1450 nm in peak γ, the electric field shown in diagrams (c), (f) is mainly concentrated at the tip of the four-pointed star. It can be seen that only the plasmon near-field coupling between the metal arrays is excited.

In order to better illustrate the superiority and rationality of the structure designed in this paper, we will further analyze the composition and design of the structure and the influence of structural parameters on absorption performance.

**Figure 4:**
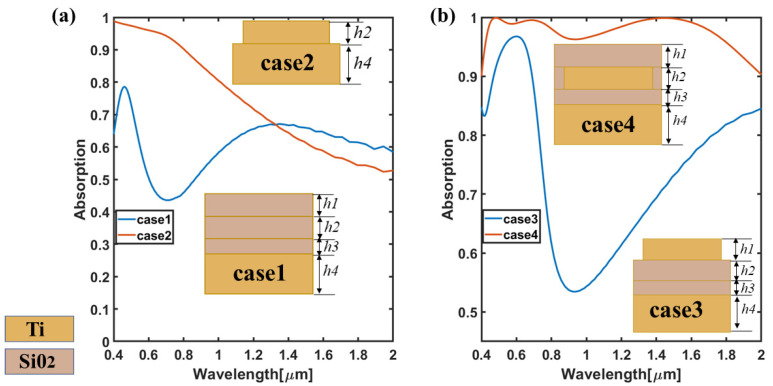
Absorption efficiency diagram under various cases. (**a**) The absorption efficiency diagram corresponding to case1 and case2; (**b**) The absorption efficiency diagram corresponding to case3 and case4.

The dimensions of each geometric parameter in the following four cases are selected from the optimal results in Figure 2. First of all, by comparing the absorption efficiency in various cases in Figure 4, we discuss the influence of the existence of the four-corner star array on the absorption efficiency. Specifically, from the absorption efficiency diagrams of case 1 and case 2, case 2 with a four-pointed star has a better absorption efficiency at 400–1200 nm and can continuously achieve broadband absorption of over 90% at 400–800 nm. However, in case 1, the absorption efficiency was lower than 80%. At the same time, it is more obvious by comparing case 1 and case 4. The existence of the four-pointed star is of great significance to the absorption efficiency, which greatly improves the absorption efficiency for the whole study wavelength. It can be seen that the broadband absorption of the absorber is mainly caused by the combination of four-pointed star array and other layers. Then, in order to explore the influence of the specific position for four-pointed array on absorption performance, we further analyze case 3 and case 4. It can be found that the position of the four-pointed star also has a great influence on the performance of absorption efficiency. Although case 3, where the four-pointed star is placed on the top, can also cause high-efficiency absorption to some extent, the absorption efficiency at 0.4–2 µm is far from the absorption effect of case 4. Therefore, through comprehensive analysis, this paper finally selects a four-layer structure, namely the nested design of Si0_2_-Ti-Si0_2_-Ti.

**Figure 5:**
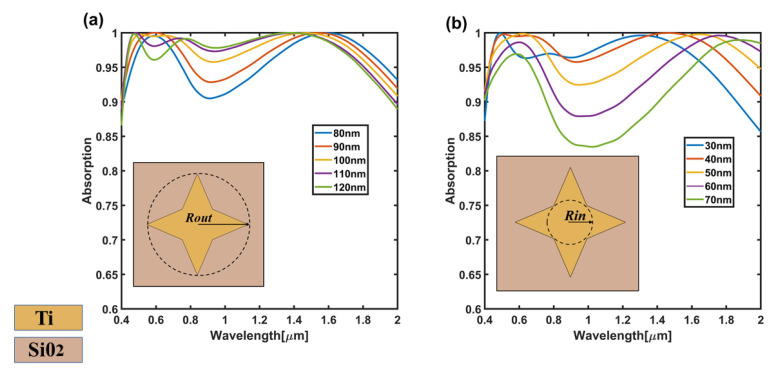
(**a**) The absorption efficiency when *Rout* changes from 80–120 nm; (**b**) The absorption efficiency when *Rin* changes from 30–70 nm.

Next, we will specifically discuss the influence of some geometric parameters on the absorption performance. When *Rout* and *Rin* are changed, the change of absorption efficiency is shown in Figure 5a. The dimensions of other geometric parameters are the same as those of the optimal structure shown in Figure 2. When *Rout* increases from 80 nm to 120 nm, it can be seen that the absorption efficiency increases significantly between 0.8–1.4 µm. In particular, the absorption efficiency of the absorption concave peak corresponding to the wavelength of 1 µm increases from the initial 0.9 to 0.97. This can be attributed to the increase of *Rout*, the strong plasmon near the field coupling caused by the tip of the four-corner star array and the enhancement of the F–P resonant cavity after area expansion. For the increase of *Rin*, it can be seen from Figure 5b that when *Rin* grows from 30 nm to 70 nm, the overall peak absorption efficiency in the long-wavelength region is red-shifted. Moreover, the absorption efficiency corresponding to the absorption peak near 1 µm decreases from 0.96 to 0.83, which is mainly attributed to the fact that the tip effect of the four-corner star is weakened with the increase of *Rin*, thus reducing the near-field coupling effect of the strong plasmon.

**Figure 6:**
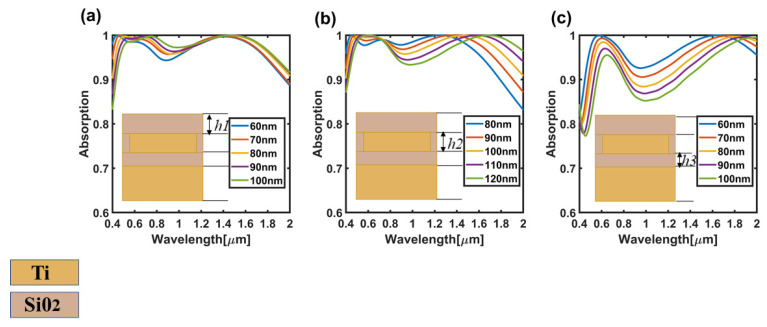
(**a**) Absorption efficiency diagram with only *h1* changing from 60–100 nm; (**b**) Absorption efficiency diagram with only *h2* changing from 80–120 nm; (**c**) Absorption efficiency diagram with only *h3* changing from 60–100 nm.

Then, the influence of different thickness of silica layer on absorption efficiency is discussed, and all the geometrical parameters of the structure adopt the size of the optimal result as shown in Figure 2, and change the height of *h1*, *h2* and *h3* successively to obtain the absorption efficiency diagram as shown in Figure 6. As shown in Figure 6, the influence of the changes in *h1* on performance was significantly lower than that of the changes in *h2* and *h3* on absorption performance. In the later stage of this paper, *h1* = 80 nm was selected as the research object. In addition, the absorption peak showed a gradual red shift. In Figure 6c, with the increase of *h3*, the absorption efficiency in almost the whole wavelength range showed a downward trend. The internal mechanism of the influence of these parameters on absorption efficiency is mainly due to the variation of plasmon resonance and the intensity of the Fabry–Perot resonant cavity. Appropriate thickness can reduce the reflection of incident light and thus enhance the absorption of incident light, but too great a thickness further affects the surface plasmon resonance of the four-corner star array [19].

**Figure 7:**
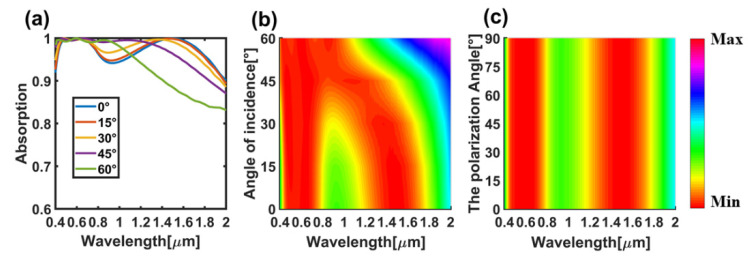
Absorption efficiency of different incident angles and polarization angles. (**a**) The absorption efficiency diagram when the incident angle is changed; (**b**) The electric field diagram when the incident angle is changed; (**c**) The electric field diagram when the polarization angle is changed.

In addition, we also tested the sensitivity of the incident angle and polarization angle of the absorber, which is also a particularly important index in practical use. Figure 7a,b show that when the incident angle varies from 0° to 60°, it keeps high-quality absorption in 0.4–1.2 µm, especially when the incident angle is 60°, as it is close to 100% perfect absorption in this band. However, at the wavelength of 1.2–2 µm, the absorption efficiency corresponding to 60° decreases significantly, while other incident angles still maintain a high absorption efficiency, which makes the average absorption efficiency corresponding to 60° lower than that corresponding to other incident angles. Fortunately, because the energy of the solar energy spectrum is small in the long wavelength region, it has little influence on the absorption of the solar energy spectrum in practice. The irrelevance of this incident angle can be mainly attributed to the complete symmetry of the absorber structure [23]. At the same time, it can be seen from Figure 7c that the intensity of the electric field at a fixed wavelength does not change with the change of the polarization angle. Therefore, the absorber designed in this paper has good independence of the incident angle and polarization angle.

**Figure 8:**
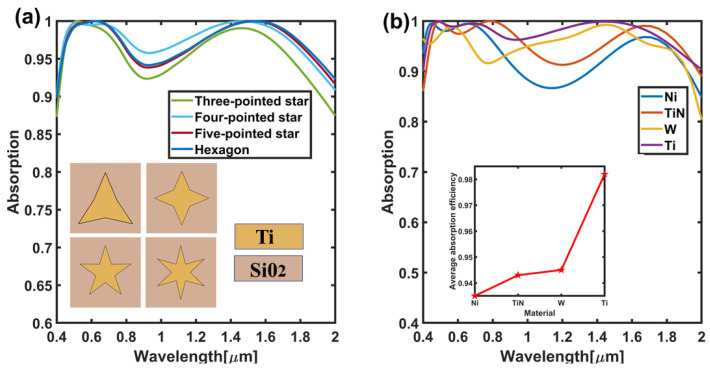
Polyhedron and different absorption efficiency of materials. (**a**) The absorption efficiency diagrams for different polygonal stars; (**b**) The absorption efficiency diagram of the four-pointed star with different materials.

Finally, in order to verify whether the absorber has certain adaptability and extensibility in terms of material and structure, we try to choose other polygons structure and high melting point materials, and then analyze the influence on absorption efficiency. As shown in Figure 8a, for several multi-star structures studied (such as triangular star, four-corner star, five-corner star and hexagonal star), the results show that different multi-corner star arrays can bring relatively high absorption efficiencies, which is mainly due to the plasmon effect under the influence of multi-corner array. Meanwhile, when analyzed for other high melting point materials such as Ni, TiN, W, etc. on the absorption efficiency of the four-corner array, the influence of the results as shown in Figure 8b show that although there are slight differences in absorption efficiency at different wavelengths, they overall have brought higher average absorption efficiency. For instance, in embedded as shown in the picture, it can be seen that the average absorption efficiency more than 93%, Ti has the best absorption efficiency.

In order to further demonstrate the excellent performance of the absorber designed in this paper, we compared the performance indexes of the absorber designed in this paper with those of other absorbers that have been published, as shown in Table 2. The results show that the structure designed in this paper is better than the same type of absorber in terms of performance index. In particular, the absorber not only ensures the high efficiency of broadband absorption, but also makes the visible light absorption band which is mainly concentrated by solar energy obtain a high efficiency absorption.

## 4. Conclusions

Overall, we put forward a kind of broadband solar energy absorber based on the four-corner array, the numerical analysis results show that the absorption apparatus with over 90% of all band absorption bandwidth (as much as 98% of the average absorption efficiency) and an overall structure of only Ti and SiO_2_ (two kinds of basic material) has good thermal stability and is easy to manufacture. The local surface plasmon resonance, strong plasmon near field coupling, Fabry–Perot (F–P) resonance and the intrinsic properties of the material contribute to the wide band absorption. At the same time, this paper studied the real absorbing properties of the absorber under the solar radiation and discussed in detail the geometric structure parameters and incident light angle and the polarization angle effect the performance of the absorption. The results show that the absorber has good insensitivity to incident angle and polarization angle, which shows that the absorption apparatus is good for environmental adaptability. Therefore, the absorber designed in this paper will be in the field of solar energy absorption, and harvesting and other fields have a promising prospect.

## Figures and Tables

**Table 1:** Related parameters of the nanostructure.

Variable Name	Significance	Variable Name	Significance
*Rout*	Circumscribed Radius	*h4*	Substrate thickness
*Rin*	Inscribed Radius	*T*	The period length of the small cell
*h1*	Top height	*θ*	Incident light angle
*h2*	The height of the four-corner star	*ψ*	Polarization angle
*h3*	Bottom height		

**Table 2:** Comparison of some absorbers with the absorber proposed in this work.

References	Material	Structure	Continuous Absorption over 90% Bandwidth/nm
Ref. [29]	Ti	Oval plate	1376
Ref. [22]	Ti, Al	cube	712.4
Ref. [20]	Ti, TiN	Double-size cross-shaped	1182
Ref. [24]	Ti, TiN	Split ring	1182
This work	Ti, SiO_2_	Four-corner star array	1600

## Data Availability

The data is available on reasonable request from the corresponding author.
